# Pesticide Removal from Aqueous Solutions by Adding Salting Out Agents

**DOI:** 10.3390/ijms141020954

**Published:** 2013-10-18

**Authors:** Fátima Moscoso, Francisco J. Deive, José M. S. S. Esperança, Ana Rodríguez

**Affiliations:** 1 Department of Chemical Engineering, University of Vigo, 36310 Vigo, Spain; E-Mails: fatimamoscoso@uvigo.es (F.M.); deive@uvigo.es (F.J.D.); 2 Instituto de Tecnologia Química e Biológica, Universidade Nova de Lisboa, Av. da República, 2780-756 Oeiras, Portugal; E-Mail: jmesp@itqb.unl.pt

**Keywords:** pentachlorophenol, ionic liquids, aqueous biphasic systems, extraction, potassium inorganic salts

## Abstract

Phase segregation in aqueous biphasic systems (ABS) composed of four hydrophilic ionic liquids (ILs): 1-butyl-3-methylimidazolium methylsulfate and 1-ethyl-3-methylimidazolium methylsulfate (C_n_C_1_im C_1_SO_4_, *n* = 2 and 4), tributylmethyl phosphonium methylsulfate (P_4441_ C_1_SO_4_) and methylpyridinium methylsulfate (C_1_Py C_1_SO_4_) and two high charge density potassium inorganic salts (K_2_CO_3_ and K_2_HPO_4_) were determined by the cloud point method at 298.15 K. The influence of the addition of the selected inorganic salts to aqueous mixtures of ILs was discussed in the light of the Hofmeister series and in terms of molar Gibbs free energy of hydration. The effect of the alkyl chain length of the cation on the methylsulfate-based ILs has been investigated. All the solubility data were satisfactorily correlated to several empirical equations. A pesticide (pentachlorophenol, PCP) extraction process based on the inorganic salt providing a greater salting out effect was tackled. The viability of the proposed process was analyzed in terms of partition coefficients and extraction efficiencies.

## 1. Introduction

Organochlorine micropollutants such as insecticides (lindane, heptachlor, DDT, *etc.*) are the subject of a great environmental and health concern since they remain in soils without significant degradation up to 30 years after their use. Among them, pentachlorophenol (PCP) is considered as an outstanding example, since it has been used in ropes, paints adhesives, brick walls, and especially as fungicide and insecticide for wood preservation. Thus, about 36 million PCP-treated utility pine poles are in service across the United States [1], which poses an undoubted environmental risk. Recent environmental water legislation has restricted or even banned the utilization of this group of pesticides, limiting the maximum allowable concentration in drinking water to 0.001 mg/L [2]. Therefore, the need to investigate efficient remediation techniques for the removal of this kind of pollutants has furthered the emergence of physico-chemical (chemical precipitation, lime coagulation, ion exchange, reverse osmosis, volatilization, photolysis, and adsorption) or biological (biosorption or biodegradation) techniques. Nevertheless, these methods present several shortcomings such as incomplete removal, sludge generation, time and energy requirements or high operating costs [3].

One of the viable alternatives for pesticides removal from aqueous solutions is the use of liquid-liquid extraction. In this sense, the strategies based on aqueous biphasic systems (ABS) where a complex competition between the polymers or salts for the water molecules [4] and specific interactions between polymers and salts have recently become an attractive option due to decisive reasons: (i) they are considered a suitable method for the separation of biomolecules [5–7], metal ions [8], and drug molecules [9]; (ii) they involve low cost and energy requirements; (iii) they entail rapid phase disengagement [10–12].

Nowadays, greener alternatives have been developed, and ionic liquids (ILs)-based ABS have been reported since 2003, when the first work on this topic was published [13]. From this year on, several papers have focused on the molecular phenomena governing IL-based ABS, varying from partition of amino acids to pharmaceuticals and biomolecules [11,14–16]. These molten salts pose an innovative alternative to conventional organic solvents, and they have opened new opportunities in extraction processes at industrial scale [17]. These molten salts are showing a tremendous growth on a diversity of fields, both fundamental and technological, making them an obvious choice for the current study. These neoteric solvents are starting to find their way into a plethora of industrial sectors, ranging from electrochemistry to biocatalysis [18]. The reason for their appeal lies in their unique properties such as, very low volatility, inflammability and recyclability [19–21]. One of the most promising characteristics of ILs is their tunability, meaning that the existence of a great number of possible combinations of cations and anions allows the design of ILs suitable for a specific industrial process. Nevertheless, although the low vapor pressure of ILs may help to reduce the air pollution, there are some ecotoxicity issues that should be taken into account prior to their use at industrial scale [22,23].

Up to date, PCP extraction was tackled only for hydrophobic ILs [24–27], so this work is the first time that hydrophilic ILs have been proposed to this end. The first purpose of this work is to provide information concerning the experimental phase segregation behavior of mixtures containing methylsulfate-based ILs, potassium inorganic salts and water. In this sense, the presence of ILs in the formation of ABS influences the possibility of changing their polarities and affinities. The proper combination cation-anion is the main advantage offered by these systems, in comparison with conventional ABS, where these properties are controlled by the amount of water in the phases. This information is vital to suitably understand separation processes and for the design and optimization of any extraction unit.

Methylsulfate-imidazolium-based ILs were chosen as models for this work since they belong to one of the most widely used families of ILs. Furthermore, they exhibit moderate viscosity, chemical stability, and low melting point temperature [28]. Additionally, phosphonium and pyridinium families were also investigated as typical commercial families of ILs. Among their specific characteristics the low viscosity of the selected ILs will favor the mass transfer and then the short time required for the ABS formation. Another important issue addressed in this work refers to the effect of the type and size of the cation for phase segregation in the presence of two inorganic salts, dipotassium hydrogen phosphate (K_2_HPO_4_), and potassium carbonate (K_2_CO_3_). The inorganic salts were selected due to their different degrees of kosmotropicity, thus licensing to map the immiscibility region. The experimental solubility curves of the hydrophilic ILs were correlated through several empirical models and the Effective Excluded Volume theory (EEV) and the results were discussed in terms of standard deviations. The proposed systems were used to elucidate their potential to extract pentachlorophenol as model persistent organic pollutant.

## 2. Results and Discussion

**Figure 1:**
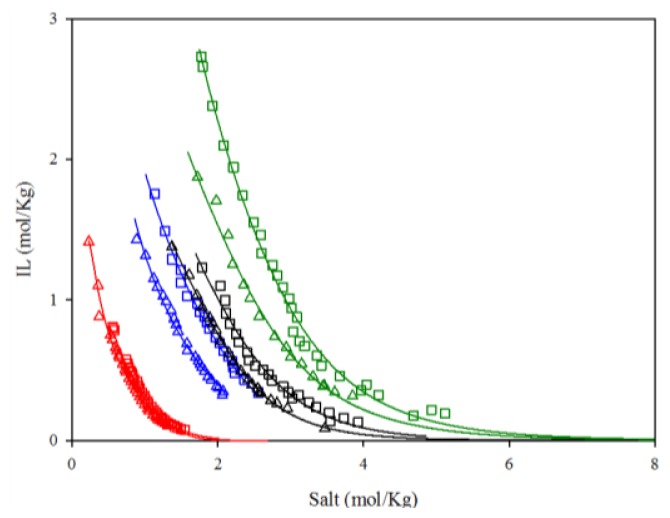
Solubility curves of the aqueous biphasic systems (ABS) formed by ILs and inorganic salts: (Δ) K_2_HPO_4_; (□) K_2_CO_3_; (**Red**) P_4441_ C_1_SO_4_; (**Blue**) C_1_Py C_1_SO_4_; (**Black**) C_4_C_1_im C_1_SO_4_; (**Green**) C_2_C_1_im C_1_SO_4_. Solid lines represent the fitting to the best empirical equation.

The experimental binodal curves for the ternary mixtures composed of aqueous solutions of the ILs (C_n_C_1_im C_1_SO_4_, *n* = 2 and 4, P_4441_ C_1_SO_4_ and C_1_Py C_1_SO_4_) and inorganic salts (K_2_CO_3_ and K_2_HPO_4_) are summarized in Table S2 and are plotted in Figure 1. The information coming from the literature [29] indicates that just experimental data related to the system composed of C_2_C_1_im C_1_SO_4_ and K_2_HPO_4_ are available and a very good agreement is obtained.

A visual inspection of the experimental curves reveals that it is possible to analyze the role of the potassium salts as phase promoters in aqueous solutions of methylsulfate-based ILs from the point of view of the inorganic cation and the molar entropy of hydration. The size and type of the IL cation was also used to understand the observed phase segregation.

Two empirical three-parameter equations [30] were used to correlate the solubility data of the selected IL-based systems. On the one hand, the characteristic exponents *n* and *m* were fixed to 0.5 and 3, respectively as it was proposed by the authors. On the other hand, a five-parameter equation was also used, as proposed recently by Deive *et al*. [31].

(1) $$w\;\;\;a\;\;\;(bw^{n}\;\;\;cw^{m})$$

(2) w         d         (w         f)         e

where *w*_1_ and *w*_2_ are the IL and potassium inorganic salt mass composition, respectively, and *a*, *b*, *c*, *d*, *e*, *f*, *n* and *m* are the fitting parameters.

The SOLVER function provided by Microsoft EXCEL was the tool used to fit the constants so that the objective function was minimized. The standard deviations were calculated by applying the following expression:

(3) $$\sigma ={\left(\sum_{\text{i}}^{{\text{n}}_{\text{DAT}}}{\left(z_{\text{exp}}-z_{\text{adjust}}\right)}^{2}/n_{\text{DAT}}\right)}^{1/2}$$

where the property values and the number of experimental and adjustable data are represented by *z* and *n*_DAT_, respectively.

The values of the coefficients obtained from the correlation of the experimental data along with the corresponding deviations are given in Tables 1 and 2. The correlation equation is also presented in Figure 1 together with the experimental data. On the basis of the deviations results it is possible to conclude that five-parameter equation is more appropriate to satisfactorily reproduce the phase diagrams of the selected IL-based ABS, no matter the IL cation and potassium inorganic salt, in agreement with the findings reported by Deive *et al.* [31].

A visual inspection of the standard deviations collected in Tables 1 and 2 allows concluding that three-parameters empirical Equation (1) is able to describe in a more suitable way the solubility data when the imidazolium-based ILs are involved. In contrast, the solubility data of phosphonium and pyridinium ILs (binodal curves closer to the origin) are better correlated by using the Equation (1). In the same line, the selected potassium inorganic salts also entail the same behavior, since a better description is attained for the strongest salting out agent (K_2_HPO_4_) by using the Equation (2).

### 2.1. Effect of Potassium Inorganic Salts on the ABS

The addition of an appropriate amount of the selected potassium based-inorganic salts allows triggering phase segregation due to the competition of the salt for the water molecules in the presence of the IL. This competition is won by the salt; and then the solubility of the IL in water subsequently decreases. The consequence associated to this phenomenon is the formation of a top phase mostly made up of IL and a bottom phase enriched in the potassium salt.

The experimental phase diagrams plotted in mol/Kg units and collected in Figure 1 indicate that different amounts of the selected salts are required for phase separation. K_2_HPO_4_ is the salt showing a stronger ability to form an immiscible area in the presence of the aqueous-IL mixtures. This salt-rank effect follows the Hofmeister series, which order ions according to their water structuring capacity. In this sense, it is possible to conclude that CO_3_^2−^ is the ion with the weakest interactions with water, thus leading to a smaller biphasic region. In contrast, the most kosmotropic HPO_4_^2−^ involves phase diagrams closer to the origin.

The molar entropy of hydration (Δ*S*_hyd_) is a novel tool to appropriately explain the observed patterns. These values were used in a previous paper [32] to ascertain the segregation potential of inorganic salts. The Δ*S*_hyd_ data published in this recent paper (HPO_4_^2−^ = −272 J/molK and CO_3_^2−^ = −245 J/molK) allows one to validate the tendency followed by the inorganic salts, thus confirming the correlation between the IL molality and the Δ*S*_hyd_.

### 2.2. Effect of IL Family and Cation Size and on the ABS

The capacity of the selected ILs to be promoted from the aqueous mixture to a top phase can be compared attending to the IL family and cation size. It can be observed that the larger immiscible area is obtained for the quaternary phosphonium based-ionic liquids, followed by pyridinium and imidazolium families (P_4441_ > C_4_Py > C_n_C_1_im). The reason for this phase segregation behavior may lie in the different charge dispersion among families. Thus, while the charge in the imidazolium family is dispersed along the aromatic moiety, the pyridinium and phosphonium cations possess the charges more concentrated on the heteroatom (nitrogen and phosphorous, respectively), as also concluded Freire and coworkers [33]. In this sense, the absence of aromaticity in the phosphonium IL corroborates its higher ability to undergo phase segregation when the inorganic salts are added.

In general terms, the charge dispersion along the imidazolium moiety confers to these ILs the ability to form hydrogen bonds, thus they can be considered as “good water solvents”. Notwithstanding this statement, the ABS will be greatly influenced by the alkyl chain length of the imidazolium-based IL. In this case, ABS segregation capacity follows the order: C_4_C_1_im C_1_SO_4_ > C_2_C_1_im C_1_SO_4_ for both potassium-based inorganic salts. The explanation behind this trend is supported by the consideration that the solubility of ILs in water is strongly influenced by their molar volume. Larger cations have been considered to better segregate two phases than smaller cations, and then, the ABS formation depends on the size of the cation [33].

The phase behavior of each system can also be analyzed in terms of the EEV theory [34], which is based on the statement that each point of the solubility curve corresponds with a geometrically saturated solution of one solute in the presence of another one. The equation to which the experimental data were fitted is:

(4) $$\text{ln}\left(V_{213}^{*}\frac{w_{2}}{M_{2}}+f_{213}\right)+V_{213}^{*}\frac{w_{1}}{M_{1}}=0$$

being $V_{213}^{*}$ the scaled EEV of the salt, *f*_213_ the volume fraction of unfilled effective available volume after tight packaging of salt molecules into the network of IL molecules in aqueous solutions, and *M*_1_ and *M*_2_, the molar mass of IL and salt, respectively.

The values of the EEV and *f*_213_ are listed in Table 3 together with the standard deviations. From the data obtained it is possible to confirm all the above-mentioned conclusions, since the phosphonium-based ILs led to the highest values of EEV, and they can be salted out more easily by the potassium inorganic salts. The same trend is checked for the imidazolium ring containing the longest alkylchain, since doubling the number of carbon atoms involved doubling the EEV values. In the same vein, the most water structuring anion (HPO_4_^2−^) led to higher values of EEV than CO_3_^2−^.

### 2.3. PCP Extraction

Once the suitability of the proposed ILs to be salted out by the selected high charge density inorganic salts has been demonstrated, the systems allowing a greater immiscibility region (those with the K_2_HPO_4_ salt) were chosen to extract PCP as model pesticide. One of the outstanding characteristics of this contaminant is its easy dissolution in water, so it is essential to reduce the concentration levels of this contaminant in wastewater. Therefore, the final step of this work consisted of analyzing the affinity of PCP for the IL-rich phase. To our knowledge, this is the first time that IL-based ABS have been used for PCP extraction. One of the useful parameters often employed for characterizing the viability of a given separation process is the partition coefficient (*K*):

(5) $$K=\frac{{\left[\text{PCP}\right]}_{\text{IL}}}{{\left[\text{PCP}\right]}_{\text{w}}}$$

where [PCP]_IL_ and [PCP]_w_ are the PCP concentration in the IL-rich phase and in the inorganic salt-rich phase, respectively.

In addition, the separation performance was analyzed in terms of extraction efficiency, *E* (%):

(6) $$E(\%)=\left(\frac{{\text{m}}_{\text{PCP}}^{\text{IL}}}{{\text{m}}_{\text{PCP}}}\right)\times 100$$

where ${\text{m}}_{\text{PCP}}^{\text{IL}}$ and m_PCP_ are the PCP mass content in the IL-rich phase and total PCP mass, respectively.

In this work, different cations paired to the same anion were investigated and the interaction with the contaminant PCP indicates significant effects on the partition coefficient into the IL phase. The values of the partition coefficients and extraction efficiencies obtained for each system are shown in Table 4.

From the partition data obtained it is clear that systems proposed are all suitable for the extraction of PCP. More specifically, the extraction capacity follows the sequence: P_4441_ C_1_SO_4_ > C_1_Py C_1_SO_4_ > C_4_C_1_im C_1_SO_4_ > C_2_C_1_im C_1_SO_4_. These results match those previously obtained for the ILs ability to form greater immiscibility regions. Furthermore, the observed pattern is in agreement with the results of selectivity reported recently by Pilli *et al.* (2012) [3], thus confirming the suitability of the phosphonium- based ILs for the implementation of this kind of remediation processes. On the other hand, the analysis of the extraction efficiencies points the ILs based on phosphonium cation as promising candidates to remove more than 99% of the pesticide present in a wastewater effluent.

The effect of the pH in the partition coefficient of PCP has been investigated with the purpose to obtain a relationship with the chemical structures of the ILs. In this sense, pH could be considered as a preliminary data to predict the segregation capacity of an organic compound in the presence of aqueous solutions of ILs. The experimental data of pH from the IL and water -rich phases indicate that the mixtures are basic (varies from 8.5 to 9.6, data listed in Table S2) for all the selected ternary mixtures. On the other hand, PCP is present as a neutral (acidic medium) or negative mono (basic medium) charged species when the pH is increased (Chemspider chemical database data [35]). Taking this into consideration, it is possible to conclude that the water content and the hydrogen bonds with the C_1_SO_4_^−^ anion govern the partition coefficients. This scenario would consequently influence the anionic form of PCP in the basic aqueous mixture, causing it to present a high hydrophobicity and also a strong electrostatic interaction with the cationic part of the phosphonium based-IL, leading to the pesticide being salted out to the charged IL-rich phase.

## 3. Experimental Section

### 3.1. Materials

C_4_C_1_im C_1_SO_4_[36] and C_1_Py C_1_SO_4_[37] were synthesized according to the procedure detailed elsewhere. C_2_C_1_im C_1_SO_4_ was purchased from Merck and P_441_ C_1_SO_4_ was kindly donated by Cytec. All the ILs were characterized by its NMR spectra and positive FABMS (FISONS VG AUTOSPEC mass spectrometer) with purity better than 99%. The water content was reduced to values less than 0.02% by means of vacuum (0.2 Pa) and moderate temperature (333.15 K) during several days. 756 Karl Fisher coulometer was used to determine the IL-water content prior to their use. Dipotassium hydrogen phosphate (K_2_HPO_4_), and potassium carbonate (K_2_CO_3_) were supplied by Sigma-Aldrich (Madrid, Spain) with purity higher than 98%, and were used as received, without further purification. The information related to the selected ILs and the potassium-based inorganic salts is collected in Table 5.

### 3.2. Solubility Curves Determination

The phase diagrams of the ABS were carried out by means of the cloud point titration method [4] at 298.15 K. A known amount of salt was added to the different IL aqueous solutions until the detection of turbidity, and then followed by the drop-wise addition of ultra-pure water until a clear monophasic region was achieved. The system was always operating under constant stirring. The ternary system compositions were determined by the weight quantification of all components within an uncertainty of ±10^−4^ g. The temperature was controlled with a F200 ASL digital thermometer with an uncertainty of ±0.01 K.

### 3.3. PCP Extraction

PCP extraction started with the addition of the pollutant (at a concentration lower than 15 mg/L) to a binary mixture (water and IL) within the miscibility region of known mass percentage and the salting out agent was added until reaching the phase segregation. The partition was carried out in graduated tubes at 298.15 K. The mixture was stirred vigorously and left to settle for 24 h to ensure a complete separation of the layers. The two phases were then carefully separated and the pesticide was quantified in both the top and bottom phases by liquid chromatography measurements. Possible interferences of the ILs were discarded by measuring each phase without PCP.

### 3.4. PCP Analysis

PCP concentrations in two phases were analyzed by reversed-phase high performance liquid chromatography (HPLC) equipped with a XDB-C8 reverse-phase column (150 × 4.6 mm i.d., 5 μm) with its corresponding guard column. The HPLC system was a HITACHI LaChrom Elite equipped with a quaternary pump (L2130) and photodiode array UV/Vis detector (280 nm). Prior to injection, the samples were filtered through a 0.45-μm Teflon filter. The injection volume was set at 0 μL, and the isocratic eluent (90:10 methanol/water) was pumped at a rate of 0.8 mL/min for 6 min.

### 3.5. pH Determination

The pH of the IL and water-rich phases was carried out at 298.15 K using a 2100 series pH meter (OAKTON instruments, Nijkerk, The Netherlands). The calibration of the pH meter was carried out with three buffers (pH values of 4.00, 7.00 and 9.00). The pH data listed in Table S1 were determined for the upper and bottom phases after the different PCP partition experiments.

## 4. Conclusions

In this work, the efficiency of several methylsulfate-based ILs as PCP extraction agents was investigated for the first time. It was demonstrated that the combination of K_2_HPO_4_ with the selected ILs involved binodal curves closer to the origin, so it was selected for the evaluation of the separation of PCP in terms of partition coefficients and extraction efficiency. The analysis of the obtained data revealed a greater ability of phosphonium-based ILs for the separation of PCP, reaching extraction efficiencies of higher than 99% and partition coefficients higher than 1000. These data are promising for further implementation of the process on a larger scale.

## Supplementary Information



## Figures and Tables

**Table 1:** Values of fitting parameters of correlation Equation (1) and standard deviation for IL + potassium inorganic salt + H_2_O at 298.15 K.

Equation 1	*a*	*b*	*c*	*n*	*m*	σ
C_2_C_1_im C_1_SO_4_ + K_2_CO_3_ + H_2_O	112.84	−0.3184	3.0 × 10^−5^			0.499
	111.63	−0.4100	3.8 × 10^−4^	0.4	2.4	0.484

C_2_C_1_im C_1_SO_4_ + K_2_HPO_4_ + H_2_O	80.00	−0.2495	2.1 × 10^−5^			0.315
	80.01	−0.2648	7.4 × 10^−5^	0.5	2.7	0.361

C_4_C_1_im C_1_SO_4_ + K_2_CO_3_ + H_2_O	80.00	−0.2969	5.0 × 10^−5^			0.464
	80.00	−0.3782	1.2 × 10^−4^	0.4	2.8	0.463

C_4_C_1_im C_1_SO_4_ + K_2_HPO_4_ + H_2_O	60.00	−0.2156	5.3 × 10^−5^			0.152
	30.61	0.0085	1.2 × 10^−3^	1.0	2.2	0.146

P_4441_ C_1_SO_4_ + K_2_CO_3_ + H_2_O	70.01	−0.3191	2.7 × 10^−4^			0.616
	71.28	−0.1445	1.0 × 10^−3^	0.9	2.4	0.243

P_4441_ C_1_SO_4_ + K_2_HPO_4_ + H_2_O	80.00	−0.3584	1.4 × 10^−4^			0.335
	33.54	0.1417	2.4 × 10^−2^	0.5	1.6	0.105

C_1_Py C_1_SO_4_ + K_2_CO_3_ + H_2_O	100.01	−0.2465	2.9 × 10^−5^			0.999
	100.07	−0.494	1.9 × 10^−2^	0.7	1.3	0.717

C_1_Py C_1_SO_4_ + K_2_HPO_4_ + H_2_O	70.00	−0.1979	2.5 × 10^−5^			0.456
	79.99	−0.3226	1.5 × 10^−3^	0.3	1.9	0.327

**Table 2:** Values of fitting parameters of correlation Equation (2) and standard deviation for IL + potassium inorganic salt + H_2_O at 298.15 K.

Equation 2	*d*	*e*	*f*	σ
C_2_C_1_im C_1_SO_4_ + K_2_CO_3_ + H_2_O	−28.38	105.10	−0.1494	0.988
C_2_C_1_im C_1_SO_4_ + K_2_HPO_4_ + H_2_O	−22.17	81.12	−4.9460	0.468
C_4_C_1_im C_1_SO_4_ + K_2_CO_3_ + H_2_O	−13.60	44.82	−10.0118	0.510
C_4_C_1_im C_1_SO_4_ + K_2_HPO_4_ + H_2_O	−21.04	74.61	−3.1855	0.400
P_4441_ C_1_SO_4_ + K_2_CO_3_ + H_2_O	−16.08	46.51	−4.4159	0.556
P_4441_ C_1_SO_4_ + K_2_HPO_4_ + H_2_O	−17.72	54.93	−3.8697	0.309
C_1_Py C_1_SO_4_ + K_2_CO_3_ + H_2_O	−21.82	78.61	−8.7861	0.617
C_1_Py C_1_SO_4_ + K_2_HPO_4_ + H_2_O	−31.56	122.26	2.8666	0.393

**Table 3:** Values of parameters of Effective Excluded Volume (EEV) and *f*_213_ for IL + potassium inorganic salt + H_2_O at 298.15 K.

	V^*^_123_/(g/mol)	*f*_213_	σ
C_2_C_1_im C_1_SO_4_ + K_2_CO_3_ + H_2_O	4.8	0.987	0.021
C_2_C_1_im C_1_SO_4_ + K_2_HPO_4_ + H_2_O	7.3	0.984	0.014
C_4_C_1_im C_1_SO_4_ + K_2_CO_3_ + H_2_O	8.0	0.983	0.046
C_4_C_1_im C_1_SO_4_ + K_2_HPO_4_ + H_2_O	12.0	0.978	0.027
P_4441_ C_1_SO_4_ + K_2_CO_3_ + H_2_O	20.0	0.973	0.028
P_4441_ C_1_SO_4_ + K_2_HPO_4_ + H_2_O	30.0	0.962	0.018
C_1_Py C_1_SO_4_ + K_2_CO_3_ + H_2_O	12.0	0.967	0.015
C_1_Py C_1_SO_4_ + K_2_HPO_4_ + H_2_O	20.0	0.954	0.011

**Table 4:** Partition coefficients *K* and extraction efficiency *E* for IL + K_2_HPO_4_ + H_2_O at 298.15 K.

System	*K*	*E* (%)
C_2_C_1_im C_1_SO_4_ + K_2_HPO_4_ + H_2_O	105	92
C_4_C_1_im C_1_SO_4_ + K_2_HPO_4_ + H_2_O	138	94
P_4441_ C_1_SO_4_ + K_2_HPO_4_ + H_2_O	1140	99
C_1_Py C_1_SO_4_ + K_2_HPO_4_ + H_2_O	183	96

**Table 5:** Materials provenance and purities.

Chemical name	Supplier	Mass fraction purity	Method of analysis
C_4_C_1_im C_1_SO_4_	Synthesized	0.99	NMR and positive FAMBS
C_2_C_1_im C_1_SO_4_	Merck	0.99	None
C_1_Py C_1_SO_4_	Synthesized	0.99	NMR and positive FAMBS
P_4441_ C_1_SO_4_	Cytec	0.99	None
K_2_CO_3_	Sigma-Aldrich	0.98	None
K_2_HPO_4_	Sigma-Aldrich	0.98	None
