# Supplementary material for: Pesticide Removal from Aqueous Solutions by Adding Salting Out Agents
Source: Int J Mol Sci. 2013 Oct 18;14(10):20954–65. doi: 10.3390/ijms141020954 (PMC3821652; doi:10.3390/ijms141020954)
Supplement: Supplementary file 1 [file ijms-14-20954-s001.pdf]

Supplementary Information

Table S1. Experimental data of pH from the IL and water -rich phases.

System	pH	
	**IL-rich phase**	**Water-rich phase**
C_2C_1im C_1SO_4 + K_2HPO_4 + H_2O	9.20	9.35
C_4C_1im C_1SO_4 + K_2HPO_4 + H_2O	9.62	9.56
P_{4441} C_1SO_4 + K_2HPO_4 + H_2O	8.49	8.76
C_1Py C_1SO_4 + K_2HPO_4 + H_2O	9.78	9.90

Table S2. Experimental solubility curves for IL + potassium inorganic salt + H_2O at 298.15 K.

$C_2C_1IMC_1SO_4$				$C_4C_1IMC_1SO_4$			
K_2CO_3		K_2HPO_4		K_2CO_3		K_2HPO_4	
w_2	w_1	w_2	w_1	w_2	w_1	w_2	w_1
8.65	43.27	17.50	24.25	15.89	19.77	15.12	21.74
13.21	32.80	20.00	22.00	18.09	17.68	17.83	18.68
13.48	32.15	22.00	19.11	18.89	16.16	19.20	16.57
14.84	29.48	23.10	16.78	19.24	14.90	20.12	15.29
16.37	26.61	24.77	14.87	19.94	13.72	21.31	14.08
17.61	24.86	25.76	13.64	20.76	12.61	21.83	13.43
18.93	22.65	27.20	11.94	21.22	11.70	22.37	12.76
20.39	20.43	29.38	9.96	22.32	10.47	23.18	11.52
21.25	19.31	30.87	8.85	22.74	9.63	24.57	10.09
21.70	17.91	31.59	8.00	23.50	8.91	25.31	9.15
22.93	16.75	32.87	7.25	24.38	8.41	26.47	8.05
23.60	15.80	34.35	6.02	25.08	7.84	27.52	7.08
24.38	14.75	35.60	5.09	25.56	7.15	28.16	6.49
25.20	13.70	35.68	5.13	26.79	6.41	28.92	5.99
25.62	12.88	36.84	4.48	27.44	5.69	29.26	5.64
26.31	12.03	38.53	4.03	27.98	5.03	29.47	5.40
26.36	10.82			28.51	5.27	30.76	4.60
27.14	9.88			29.63	4.43	31.51	4.22
27.75	9.36			30.47	3.83	32.79	3.61
29.07	8.38			31.75	3.19	37.20	1.30
29.71	7.44			32.20	2.19		
31.59	6.29			33.18	2.56		
33.72	4.84			34.49	2.03		
33.91	5.36						
35.19	4.33						
38.41	2.30						
39.40	2.79						
40.41	2.42						

Table S2. *Cont.*

$P_{4441}C_1SO_4$				$C_1PyC_1SO_4$			
K_2CO_3		K_2HPO_4		K_2CO_3		K_2HPO_4	
w_2	w_1	w_2	w_1	w_2	w_1	w_2	w_1
6.34	28.45	4.02	46.44	15.72	36.75	15.44	29.91
7.78	26.37	6.26	36.25	17.67	31.14	17.61	27.53
7.80	26.55	6.50	28.94	18.92	26.98	19.52	24.12
8.03	25.78	9.21	24.72	20.63	25.43	20.42	22.87
8.93	22.18	9.69	23.63	20.67	23.49	21.82	21.49
9.38	21.31	10.45	21.81	21.87	21.51	22.51	20.73
9.91	19.96	10.76	20.97	23.86	20.34	23.73	19.37
10.23	18.91	11.18	19.81	24.19	19.13	24.37	18.12
10.53	18.04	11.34	19.41	25.10	18.43	24.91	17.25
10.74	17.05	12.09	17.86	25.57	17.63	25.39	16.25
11.10	16.36	12.72	16.66	26.02	16.62	27.47	14.42
11.31	15.86	12.98	16.03	27.17	15.40	27.60	13.40
11.54	15.15	13.67	14.60	27.95	14.49	29.49	12.38
11.85	14.26	14.14	13.72	28.69	13.35	30.23	11.82
12.41	13.71	14.68	12.82	29.63	12.54	30.64	11.35
12.48	12.85	15.17	11.95	30.35	11.79	31.05	10.88
12.87	12.28	15.65	11.11	30.59	10.89	31.71	10.34
13.26	11.19	16.18	10.21	30.82	10.11	32.55	9.49
13.73	10.37	16.72	9.42	32.68	9.03	33.26	9.02
13.87	9.75	17.35	8.43	35.32	7.15	34.57	8.06
14.34	9.07	18.10	7.45			34.98	7.80
14.53	8.45	18.42	7.00			36.10	7.29
15.10	7.75	19.39	6.00			35.93	6.73
15.53	7.13	20.05	5.34				
15.99	6.55	20.54	4.96				
16.33	6.01	21.02	4.62				
16.84	5.47	21.60	4.23				
17.22	5.08	22.25	3.89				
17.67	4.56	22.90	3.40				
18.16	4.10						
18.80	3.67						
19.47	3.26						
20.06	2.91						
20.71	2.61						
21.38	2.35						

© 2013 by the authors; licensee MDPI, Basel, Switzerland. This article is an open access article distributed under the terms and conditions of the Creative Commons Attribution license (http://creativecommons.org/licenses/by/3.0/).
